# The effects of a rise in cigarette price on cigarette consumption, tobacco taxation revenues, and of smoking-related deaths in 28 EU countries-- applying threshold regression modelling

**DOI:** 10.1186/s12889-017-4685-x

**Published:** 2017-09-21

**Authors:** Chun-Yuan Yeh, Christian Schafferer, Jie-Min Lee, Li-Ming Ho, Chi-Jung Hsieh

**Affiliations:** 1 Department of International Trade, Overseas Chinese University, Taichung, Taiwan; 2 Department of Shipping and Transportation Management, National Kaohsiung Marine University, 142, Hai-Chuan Rd. Nan-Tzu, Kaohsiung, Taiwan; 3 Department of Marine Leisure Management, National Kaohsiung Marine University, Kaohsiung, Taiwan; 4 Department of Finance, National Changhua University of Education, Changhua, Taiwan

**Keywords:** Cigarette price, Cigarette consumption, Threshold regression model-Smoking-attributable mortality, European Union

## Abstract

**Background:**

European Union public healthcare expenditure on treating smoking and attributable diseases is estimated at over €25bn annually. The reduction of tobacco consumption has thus become one of the major social policies of the EU. This study investigates the effects of price hikes on cigarette consumption, tobacco tax revenues and smoking-caused deaths in 28 EU countries.

**Methods:**

Employing panel data for the years 2005 to 2014 from Euromonitor International, the World Bank and the World Health Organization, we used income as a threshold variable and applied threshold regression modelling to estimate the elasticity of cigarette prices and to simulate the effect of price fluctuations.

**Results:**

The results showed that there was an income threshold effect on cigarette prices in the 28 EU countries that had a gross national income (GNI) per capita lower than US$5418, with a maximum cigarette price elasticity of −1.227. The results of the simulated analysis showed that a rise of 10% in cigarette price would significantly reduce cigarette consumption as well the total death toll caused by smoking in all the observed countries, but would be most effective in Bulgaria and Romania, followed by Latvia and Poland. Additionally, an increase in the number of MPOWER tobacco control policies at the highest level of achievment would help reduce cigarette consumption.

**Conclusions:**

It is recommended that all EU countries levy higher tobacco taxes to increase cigarette prices, and thus in effect reduce cigarette consumption. The subsequent increase in tobacco tax revenues would be instrumental in covering expenditures related to tobacco prevention and control programs.

## Background

### Smoking prevalence and tobacco control

In 2017, every fourth EU citizen smoked, and the annual death toll from smoking was approximately 700,000 [1]. EU public healthcare expenditure on treating smoking and attributable diseases is estimated at over €25bn annually, constituting a considerable burden on public health care systems. Moreover, €8bn is reportedly lost annually in productivity from deaths, absenteeism and early retirement linked to smoking [2].

The reduction of tobacco consumption has thus become one of the major social policies of the EU and the necessity of implementing the six MPOWER tobacco control policies proposed by the World Health Organisation (WHO) in 2008: (i) increases in the tobacco tax; (ii) monitoring of tobacco usage; (iii) support for quitters; (iv) creation of a smoking-free environment; (v) warning against the dangers of tobacco; (vi) and banning tobacco advertising, promotion and sponsorship. According to Levy et al., if 41 countries across the world had implemented at least one MPOWER policy between 2007 and 2010, the number of smokers would have been cut by 14.8 million, and 7.4 million would have avoided death caused by smoking [3]. In particular, increased tobacco taxation would have saved 3.5 million people, suggesting that taxation would have been the most effective single intervention to reduce demands for cigarettes by raising cigarette prices [3]. The relationship between tobacco price and consumption is also illustrated by the fact that EU countries with lower cigarette prices tend to have higher rates of smoking [4]. Moreover, unlike other policy tools, such as bans on tobacco advertising, taxation not only effectively decreases tobacco consumption but also has the beneficial side effect of increasing national tax revenues [5, 6].

### Price elasticities and cigarette demand

The effectiveness of tax increases on cigarette consumption is mainly determined by cigarette price elasticity. Although numerous studies have demonstrated that cigarette price elasticities of low- and middle-income are higher than in high-income countries [7], other studies have shown that price elasticity in several developing countries were similar to those of developed countries [8, 9]. Differences in cigarette price elasticities may have been caused by applying different demand functions, information patterns, and estimation methods [10]. Therefore, employing the same demand function, information pattern, and estimation method can facilitate a standardized comparison of the price elasticity of demand for cigarettes among different countries.

Studies have also shown that there are geographical variations in smoking behaviour [11, 12]. In Eastern European countries, such as Slovenia, Romania and Slovakia, tobacco prevalence in rural and remote areas is higher than in urban areas (World Bank. World Development Indicators (WDI)), whereas in Western European countries, such as Germany, Sweden, Finland and Denmark, the opposite has been reported [13]. Smokers living in rural and remote areas tend to have a lower social and economic status and are more sensitive to price fluctuations [14, 15]; hence, this group reportedly is more likely to opt for lower-priced products, or consider cessation [16, 17]. Moreover, cigarette prices in Eastern European countries are considerably lower than in other parts of Europe, leading to increased illicit trading in cigarettes and to subsequent changes in consumption patterns throughout the European Union [18].

Numerous previous studies have adopted a linear model to estimate cigarette demand structure [4, 9, 19]. However, this estimation method might fail to fully show the price-volume relationship. Huang and Yang estimated the cigarette demand relationship in all states across the US and found that there were income threshold effects in the demand for cigarettes [20], which indicates that there are differences in the cigarette price elasticity of demand at different income thresholds.

### The goals of this study

This study employed threshold regression modelling and used income as a threshold variable to estimate the price elasticity of cigarette demand. Furthermore, a cigarette price increase of 10% was used to analyse the effects of price increases on cigarette consumption, tobacco taxation, and the death toll of smoking. The findings of this study may serve as an important reference for EU health management authorities to revise tobacco prevention and control policies.

## Methods

### Study design and data

In this study, data of all 28 EU countries were collected to construct a cigarette demand structure model. One dependent variable and five independent variables were considered. Per capita cigarette consumption for those aged 15 and over was chosen as the dependent variable. Independent variables comprised cigarette prices, cigarette prices in Eastern European countries, gross national income (GNI), rural population, and the number of MPOWER measures implemented at the highest level of achievement.

Data regarding cigarette consumption, cigarette prices, and cigarette prices in Eastern European countries were extracted from the 2005–2014 Euromonitor International market research database [21]. Euromonitor International is recognized as a leading independent provider of global business intelligence, specialized in creating worldwide data and analysis on consumer products and services. Consumption of cigarette products was calculated based on annual cigarette consumption per capita for those aged 15 and over. The retail price for a pack of cigarettes in each country was calculated by dividing cigarette sales revenues by cigarette consumption, which was further deflated using consumer price indexes.

Cigarette price in Eastern European countries refers to the combined average cigarette price in Estonia, Latvia, Lithuania, Poland, Czech Republic, Slovakia, Hungary, Romania, Slovenia, Croatia and Bulgaria.

GNI per capita data were converted to US dollars using the World Bank Atlas method [22], divided by the midyear population, and deflated based on consumer price indexes. Required data were retrieved from the World Bank’s database. In this study, the ratio of the rural population to the total population was used in the analysis. Rural population refers to the number of people living in rural areas as defined by the National Statistical Offices and was calculated as the difference between the total population and the urban population. Data on the ratio of the rural population to the total population are World Bank estimates [22] and based on the United Nations, World Urbanization Prospects [23].

As to the number of MPOWER measures implemented at the highest level of achievement by each country in each year, figures for the years 2007 to 2014 were taken from the 2015 WHO report on global tobacco epidemic [17]. Data for the years 2005, 2006 and 2015 were unavailable and treated as missing data in the analysis.

### Data characteristics

Table 1 shows the list of variables used in the analysis and the data characteristics. In 2014, per capita cigarette consumption in the 28 EU countries for adults aged 15 years and over was the highest in Slovenia at 2098 cigarettes, followed by the Czech Republic (1720 cigarettes), Austria (1631 cigarettes), Greece (1552 cigarettes), and Romania (1501 cigarettes); those of the other EU countries were all less than 1500 cigarettes. In 2014, the average real retail price was the highest in United Kingdom at US$9.48 per pack, followed by Ireland (US$9.04). In addition, the average highest number of MPOWER measures implemented in Ireland, Spain, and the United Kingdom was 3, followed by Belgium, Bulgaria, Denmark, Greece, Malta, and the Netherlands at 2; whereas in the remaining EU countries the number of measures were below 2.

**Table 1:** Comparison of cigarette consumption, retail prices and gross national income from 2005 to 2014 in the European Union

Countries	Per capita legal cigarette consumption of population aged over 15 (No. cigs)			Real retail price of a pack of legal cigarettes (US$)			Real GNI per capita (US$) Consumer price index (2005 = 100)			The number of MPOWER measures at the highest level of achievement in 2014	Share of total taxes in the retail price in 2014
	2005	2014	Change (2005 ~ 2014)	2005	2014	Change (2005 ~ 2014)	2005	2014	Change (2005 ~ 2014)		
Austria	1688	1631	−3.38%	3.27	4.59	40.37%	38,500	41,765	8.48%	1	74.00%
Belgium	1156	966	−16.44%	4.5	5.24	16.44%	37,850	39,561	4.52%	2	75.92%
Bulgaria	2750	1282	−53.38%	0.8	1.6	100.00%	3760	5261	39.92%	2	82.65%
Croatia	1735	1330	−23.34%	1.69	2.74	62.13%	9870	10,471	6.09%	0	75.26%
Czech Republic	2183	1720	−21.21%	1.77	2.79	57.63%	12,380	15,246	23.15%	1	77.42%
Cyprus	1371	1400	2.12%	2.06	2.72	32.04%	21,490	22,597	5.15%	0	77.47%
Denmark	1472	1080	−26.63%	4.06	5.15	26.85%	49,620	52,632	6.07%	2	74.75%
Estonia	1481	1480	−0.07%	1.25	2.27	81.60%	9710	13,436	38.37%	1	77.24%
Finland	954	789	−17.30%	4.05	5.44	34.32%	40,100	40,954	2.13%	1	81.53%
France	898	682	−24.05%	5	7.07	41.40%	36,000	37,487	4.13%	1	80.3%
Germany	1175	980	−16.60%	4.49	5.23	16.48%	35,880	41,335	15.20%	1	72.9%
Greece	3016	1552	−48.54%	2.46	3.3	34.15%	22,510	18,212	−19.09%	2	79.95%
Hungary	1366	936	−31.48%	1.52	1.88	23.68%	10,430	9202	−11.77%	1	77.26%
Ireland	1364	685	−49.78%	6.01	9.04	50.42%	44,500	32,362	−27.28%	3	77.8%
Italy	1569	1147	−26.90%	3.57	4.9	37.25%	32,390	29,248	−9.70%	1	75.68%
Latvia	2089	892	−57.30%	0.81	2.02	149.38%	7360	10,293	39.85%	1	76.89%
Lithuania	1079	965	−10.57%	1.26	2.42	92.06%	7550	11,421	51.27%	1	75.76%
Luxembourg	1110	854	−23.06%	4.57	4.63	1.31%	70,340	62,685	−10.88%	1	70.24%
Malta	1661	1048	−36.91%	2.76	3.77	36.59%	14,380	19,645	36.61%	2	74.63%
Netherlands	837	659	−21.27%	3.13	5.92	89.14%	42,390	43,804	3.34%	2	73.4%
Poland	1926	1136	−41.02%	1.17	2.52	115.38%	7330	10,858	48.13%	1	80.29%
Portugal	1754	852	−51.43%	2.29	4.62	101.75%	18,550	18,342	−1.12%	1	74.51%
Romania	1604	1501	−6.42%	0.23	0.55	139.13%	3930	6190	57.51%	1	75.41%
Slovakia	1136	1381	21.57%	1.26	1.83	45.24%	11,280	14,410	27.75%	1	81.54%
Slovenia	2281	2098	−8.02%	1.54	2.47	60.39%	18,440	19,217	4.21%	1	80.41%
Spain	2212	911	−58.82%	2.79	4.09	46.59%	25,930	24,424	−5.81%	3	78.09%
Sweden	2212	666	−69.89%	4.93	6.37	29.21%	45,350	54,339	19.82%	1	68.84%
United Kingdom	843	568	−32.62%	7.87	9.48	20.46%	41,150	34,194	−16.90%	3	82.16%

### Empirical specification and analysis

To calculate cigarette price elasticity, a cigarette demand structure model was constructed using cigarette consumption as the dependent variable and cigarette price, cigarette prices in Eastern Europe countries, GNI, rural population, and the highest number of MPOWER measures implemented as explanatory variables. Cigarette price elasticity was estimated with income as the threshold variable using the threshold regression model of panel data from Hansen [24].

The baseline cigarette demand structure model of the 28 EU countries is as follows:

1 $$ \ln {C}_{it}={\beta}_{1i}+{\beta}_2\ln {P}_{it}+{\beta}_3\ln {GNI}_{it}+{\beta}_4{Rural}_{it}+{\beta}_5{MP}_{it}+{\beta}_6\ln {NeiP}_{it}+{\varepsilon}_{it} $$

Where,

*C*_*it*_: the annual cigarette consumption per capita in the population aged 15 years and over in country *i* in year *t.*

*P*_*it*_: the cigarette price per cigarette in country i in year t.

*GNI*_*it*_: the per capita national income in country i in year t.

*Rural*_*it*_: the rural population percentage in country i in year t.

*MP*_*it*_: the highest number of MPOWER measures implemented in country i in year t.

*NeiP*_*it*_: cigarette prices per cigarette in Eastern European country i in year t.

Formula (1) is the traditional constant-elasticity log-linear demand model, but the influence of cigarette prices on cigarette consumption may not be limited to a single pattern. That is, there may also be non-linear structural relationships, such as income threshold effects on the demand for tobacco products [20]. We thus used income as the threshold variable in the threshold regression model to estimate the elasticity of cigarette prices and to simulate the effects of price fluctuations. One feature of a threshold regression model is that threshold variables are ordered so as to be a structure breakpoint of regime variation. The estimated reference points are divided into different regimes by variable value, which is greater or smaller than the threshold value.

The panel threshold regression model is often “de-meaned” first, in order to eliminate the individual effect *β*_*i*_ [24]. If our baseline model contains three regimes of national incomes that are conditional on two threshold values, Eq. 1 can be derived as

2 $$ {\displaystyle \begin{array}{l}\ln {C}_{it}^{\ast }={\beta}_{21}\ln {P}_{it}^{\ast}\left( GNI\le {\gamma}_1\right)+{\beta}_{22}\ln {P}_{it}^{\ast}\left({\gamma}_1\le GNI\le {\gamma}_2\right)+{\beta}_{23}\ln {P}_{it}^{\ast}\left( GNI>{\gamma}_2\right)+\\ {}\kern3.12em {\beta}_{31}\ln {GNI}_{it}^{\ast}\left( GNI\le {\gamma}_1\right)+{\beta}_{32}\ln {GNI}_{it}^{\ast}\left({\gamma}_1\le GNI\le {\gamma}_2\right)+{\beta}_{33}\ln {GNI}_{it}^{\ast}\left( GNI>{\gamma}_2\right)\\ {}\kern2.84em +\kern0.72em {\beta}_4{Rural}_{it}^{\ast }+{\beta}_5{MP}_{it}^{\ast }+{\beta}_6\ln {NeiP}_{it}^{\ast }+{\varepsilon}_{it}^{\ast}\end{array}} $$

Where ${\overline{C}}_i={T}^{-1}{\sum}_{t=1}^T{C}_{it}$; ${C}_{it}^{\ast }={C}_{it}-\overline{C_i}$; ${P}_{it}^{\ast }={P}_{it}-\overline{P_i}$; ${GNI}_{it}^{\ast }={GNI}_{it}-{\overline{GNI}}_i$; ${Rural}_{it}^{\ast }={Rural}_{it}-{\overline{Rural}}_i$; ${MP}_{it}^{\ast }={MP}_{it}-{\overline{MP}}_i$; ${NeiP}_{it}^{\ast }={NeiP}_{it}-{\overline{NeiP}}_i$; ${\varepsilon}_{it}^{\ast }={\varepsilon}_{it}-{\overline{\varepsilon}}_i$; and γ_1_ and γ_2_ are the two threshold values that control *GNI*_*it*_. We use ordinary least squares (OLS) to estimate Eq. 2. The selection of threshold variables in the empirical model can be determined by economic theory or statistical testing. In the process of statistical testing, the null hypothesis (H_0_: *β*_*i*_ are all the same) maintains that a traditional log-linear model is sufficient. In this article, we applied the likelihood ratio to test the nonlinear relationship. In such cases, further testing is required to determine single, double or triple thresholds. In order to avoid heteroskedasticity, the inconsistency of standard errors caused by serial correlations and heterogeneity of the residual terms, consistent correction was performed according to White (1980) [25]. Statistical software packages Gauss and Stata were used to perform the analysis.

To determine the effects of cigarette price hikes on cigarette consumption, cigarette consumption in 2015 was set as the baseline for this study. We introduced 10% increments in cigarette prices to simulate changes in future cigarette consumption based on the cigarette price elasticity estimated in this study. Changes in tobacco tax revenues were calculated based on changes in consumption due to price increases.

The number of averted smoking-attributable deaths (SADs) derived from the simulated impact of price increments on the reduction in smokers and was adjusted for the fact that smoking cessation still carries considerable risks of early death [26]. The applied mortality adjustment factors were calculated for each country surveyed, assuming that 95, 75, 70, 50 and 10% of those who ceased smoking when aged 15 to 29, 30 to 39, 40 to 49, 50 to 59 and at least 60 years, respectively, would remain unaffected by their previous smoking habits [27]. Data on population stratification were extracted from the Eurostat database.

## Results

### Regression results

Table 2 shows the test results of the threshold effect. For the observed EU countries, the test for one threshold had a value of 43.66, suggesting that price elasticities may indeed fluctuate as a result of changing income levels. The panel threshold model seems thus to be more appropriate to apply in this study than the traditional panel data model. Next, the value of the LR with two thresholds was 36.61, which allowed us to reject the null hypothesis that there was no presence of a threshold effect at the 10% significance level. Furthermore, there were two significant income threshold values in cigarette prices in the EU at US$5418 and at US$8385. As the sum of squared errors (SSE) of the double-threshold model (0.311) is lower than that of the single-threshold model (0.355), the double-threshold model seems to be more appropriate.

**Table 2:** Threshold model estimates

	Dependent variable: (lnC^*^_it_)	
	Single-income threshold model	Double-income threshold model
Independent variables		
ln P^*^_it_		
Regime1(GNIit≦US$5418)	−1.315 ^b^ (−0.737,-1.893)	−1.227 ^b^(−0.667,-1.787)
Regime2(GNIit > US$5418)	−0.638 ^b^ (−0.427,-0.849)	
Regime2(US$5418 < GNIit≦US$8385)		−0.829 ^b^ (−0.628,-1.030)
Regime3(GNIit > US$8385)		−0.503 ^b^ (−0.291,-0.715)
lnGNI^*^_it_		
Regime1(GNIit≦US$5418)	0.332 ^b^ (0.596,0.069)	0.282 ^b^ (0.530,0.033)
Regime2(GNIit > US$5418)	0.596 ^b^ (0.827, 0.366)	
Regime2(US$5418 < GNIit≦US$8385)		0.423 ^b^ (0.685,0.261)
Regime3(GNIit > US$8385)		0.576 ^b^ (0.787,0.336)
MP^*^_it_	−0.021 ^b^ (−0.006,-0.035)	−0.017 ^b^ (−0.003,-0.032)
Rural^*^_it_	1.637 ^b^ (3.006,0.269)	1.616 ^b^ (2.830,0.402)
lnNeiP^*^_it_	0.031 (0.184,-0.122)	−0.057 (0.083,-0.197)
Observations	252	252
Number of country	28	28
Sum of square error (SSE)	0.355	0.311
Test for one threshold (income threshold) γ1	43.66 ^a^ (5418)	
Test for two thresholds (income threshold) γ2		36.61 ^a^ (6494,32,040)

Regime 1 countries: Bulgaria, Romania

Regime 2 countries: Latvia, Poland

Regime 3 countries: Austria, Belgium, Croatia, Cyprus, Czech Republic, Denmark, Estonia, Finland, France, Germany, Greece, Hungary, Ireland, Italy, Lithuania, Luxembourg, Malta, Netherlands, Portugal, Slovakia, Slovenia, Spain, Sweden, United Kingdom

*P* ^*^ legal cigarette price, *GNI* ^*^ per capita national income, *Rural* ^*^ rural population %, *MP* ^*^ the number of MPOWER measures at the highest level of achievement. Confidence intervals are shown in parentheses

^a^Statistically significant at 10% level

^b^Statistically significant at 5% level

This study thus used three income regimes with different GNI per capita in the analysis. As illustrated in Table 3, Bulgaria and Romania were assigned to the first, Latvia and Poland to the second, and the remaining 24 EU countries to the third income regime.

**Table 3:** Impact of cigarette price increases by 10% on cigarette consumption, tax revenue, and reduction in smoking-attributable deaths

Countries	Average real price per pack in 2015 (US$)	Increase in price due to price scenarios increase 10% (US$)	Estimated cigarette price elasticities	Change in cigarette consumption (%)	Change in cigarette consumption (million packs)	Change in cigarette tax revenue (%)	Change in cigarette tax revenue (million US$)	Reduction in no. of smokers due to price increases	Reduction in no. of SADs^b^
Regime1(GNIit≦US$5418)									
Bulgaria, Romania	1.15	0.115	−1.227	−12.27	−230.15	−1.41	−14.4	588,614	251,860
Regime2 (US$5418 < GNIit≦US$8385)									
Latvia, Poland	2.32	0.232	−0.829	−8.29	−206.84	3.15	139.5	489,546	210,061
Regime3 (GNIit > US$8385)									
EU24 countries (Austria, Belgium, Croatia, Czech Republic, Cyprus, Denmark, Estonia, Finland, France, Germany, Greece, Hungary, Ireland, Italy, Lithuania, Luxembourg, Malta, Netherlands, Portugal, Slovakia, Slovenia, Spain, Sweden, United Kingdom)	4.63	0.463	−0.503	−5.03	−1019.48	7.03	6523	3,123,560	1,419,959
All EU28 countries	4.15	0.415	−0.587	−5.87	−1310.09	6.76	6644	4,201,720	1,879,880

*SADs* smoking-attributable death

^a^The reduction in the number of smokers equals the reduction in cigarette consumption as a result of price increases multiplied by the number of adult smokers

^b^The number of SADs averted was calculated according to Goodchild et al. [6]: Reduction in SADs = Reduction in no. of smokers multiplied by the corresponding mortality adjustment factor

### Elasticity estimates

Our model showed differences in cigarette price elasticity figures of each income threshold (Table 2). When GNI per capita was lower than US$5414 (Regime 1), cigarette price elasticity was the highest at −1.227 and income elasticity the lowest at 0.282. When GNI per capita was between US$5414 and US$8385 (Regime 2), cigarette price elasticity reached −0.829 and income elasticity 0.423. When GNI per capita was higher than US$8385 (Regime 3), cigarette price elasticity was the lowest at −0.503 and income elasticity the highest at 0.576. In addition, among the 28 EU countries cigarette prices in Eastern European countries ($$ {NeiP}_{it}^{\ast } $$) had a negative impact on cigarette consumption (−0.057), indicating that lower cigarette prices in Eastern European countries led to higher domestic consumption.

The number of MPOWER measures implemented at the highest level of achievement had a negative and statistically significant impact on cigarette consumption. Moreover, living in rural areas had a positive and statistically significant impact on cigarette consumption.

### Effect of cigarette prices on cigarette consumption, tobacco tax revenue, and smoking-related deaths

Results of the administered price simulation showed that increases in cigarette prices (10%) would reduce cigarette consumption the most in Bulgaria and Romania (per pack average price increase US$0.115; consumption reduced: 12.27%), followed by Latvia, and Poland (per pack average price increase US$0.232; consumption reduced:8.29%). The largest group of EU countries (in the following referred to as EU 24) had a smaller reduction in cigarette consumption (consumption reduced: 5.03%) (Table 3).

Furthermore, the administered price simulation exhibited different effects on tax revenues among the three income groups. Bulgaria and Romania had a decrease in tax revenues (−1.41%), whereas the EU24 countries had the highest average increase in tax revenues (7.03%). Latvia and Poland, on the other hand, showed the lowest average increase in tax revenues (3.15%) among all EU countries.

The simulated tax increase showed a significant impact on the number of averted smoking-attributable deaths (SADs) in all EU countries, but low-income countries were much more affected by the policy measure than the richer EU24 countries. According to the simulation, Bulgaria and Romania would avert over 251,860 deaths, followed by Latvia and Poland with 210,061 people, whereas the number of averted SADs would reach about 1.4 million in the EU24 zone.

## Discussion

According to the results of this study, the price elasticity of cigarette demand in the 28 countries comprising the EU ranged from −0.503 to −1.227. Countries with a GNI per capita lower than US$5418 had the highest cigarette price elasticity (−1.227). Countries with a GNI per capita higher than US$5418 had lower cigarette price elasticities. These findings were similar to International Agency for Research on Cancer [28], who showed that low- and middle-income countries had a higher price elasticity. The results of this study thus further emphasise the importance of using economic measures as an intervention tool, especially in countries such as Bulgaria, and Romania, to reduce smoking.

In addition, this study estimated that income elasticities of cigarette demand ranged from 0.282 to 0.576. Previously reported estimates ranged between 0.3 and 0.4 [4]. Despite differences in the size of the observed effect, elasticity figures suggest that income growth may have promoted cigarette consumption in the observed EU countries. That is, the effects of price increases on consumption might be offset by income growth in countries with a GNI per capita above US$8385, where estimated cigarette price elasticity was lower than income elasticity. Thus, almost all EU countries would have to increase their cigarette prices substantially to reduce the increase in cigarette consumption that might be caused by income growth offset effects.

This study found that an average price increase of 10% throughout the EU would lead to an average increase in revenues by about 6.76%, which is consistent with previously reported results [29]. Moreover, results of this study showed that the average tobacco taxation benefit of all EU countries significantly increased by 6644 million US$ as a result of rising cigarette prices. In the future, increased cigarette prices in all EU countries are likely to reduce further the demand for cigarettes, and the appreciable increase in tobacco taxation revenues could be spent on the prevention and control of cigarette-related diseases.

Additionally, this study revealed a negative relationship between the number of MPOWER measures at the highest level of achievement and cigarette consumption, which implies that t the implementation of MPOWER measures can reduce cigarette consumption in the 28 EU countries. In most countries, however, the number of MPOWER measures at the highest level of achievement was below 2, That is, the number of implemented MPOWER tobacco control policies among EU countries was rather small. Therefore, efforts have to be made to implement far more MPOWER tobacco control policies as to achieve greater effects in reducing cigarette consumption as a whole.

This study showed that price increases in Bulgaria, and Romania had the greatest effects on reducing cigarette consumption among all EU countries, while experiencing a comparatively high prevalence of smoking [30]. In spite of gradual increases in tobacco taxes as to control tobacco consumption in recent years, cigarette prices in Bulgaria, and Romania have still remained comparatively low [2, 31]. If these two countries continued to increase cigarette prices and the number of MPOWER tobacco control policies, current achievements could be significantly enhanced and funding for prevention and control programmes substantially be improved by larger taxation revenues. Transnational information was analysed in this study. However, the integrated analysis of transnational information may lead to incorrect inferences because different countries have different cigarette consumption structures. We thus suggest that all countries establish a cigarette consumption database and market-monitoring mechanism to undertake long-range tracking and analysis.

Illicit trade of tobacco products has not been included in the study, as reliable data could not be obtained for all countries. Moreover, data on cigarette consumption analysed in this study refer to factory-made (FM) cigarettes.Roll-your-own (RYO) tobacco products have become popular in the EU in recent years and may influence consumption behaviour. Further research on price effects may thus address the issue of illicit trade and RYO cigarette use.

## Conclusion

This study estimated that price elasticities of cigarette demand ranged from −0.503 to −1.227. These figures were negative, indicating that cigarette consumption or demand in 28 EU countries were inelastic during the study period. It is recommended that the 28 EU countries should levy a higher tobacco tax to increase cigarette prices, thus reducing cigarette consumption. The subsequent increase in tobacco tax revenues would be instrumental in covering expenditures related to tobacco prevention and control programs.
